# Investigating the role of human frontal eye field in the pupil light reflex modulation by saccade planning and working memory

**DOI:** 10.3389/fnhum.2022.1044893

**Published:** 2022-11-17

**Authors:** Tzu-Yu Hsu, Hsin-Yi Wang, Jui-Tai Chen, Chin-An Wang

**Affiliations:** ^1^Graduate Institute of Mind, Brain, and Consciousness (GIMBC), Taipei Medical University, Taipei City, Taiwan; ^2^Brain and Consciousness Research Center (BCRC), TMU-Shuang Ho Hospital, New Taipei City, Taiwan; ^3^Department of Anesthesiology, School of Medicine, College of Medicine, Taipei Medical University, Taipei City, Taiwan; ^4^Department of Anesthesiology, Shuang Ho Hospital, Taipei Medical University, New Taipei City, Taiwan; ^5^Institute of Cognitive Neuroscience, College of Health Science and Technology, National Central University, Taoyuan City, Taiwan; ^6^Cognitive Intelligence and Precision Healthcare Research Center, National Central University, Taoyuan City, Taiwan

**Keywords:** TMS, cTBS, superior colliculus, pupillometry, spatial attention, pseudoneglect, frontal eye field

## Abstract

The pupil constricts in response to an increase in global luminance level, commonly referred to as the pupil light reflex. Recent research has shown that these reflex responses are modulated by high-level cognition. There is larger pupil constriction evoked by a bright stimulus when the stimulus location spatially overlaps with the locus of attention, and these effects have been extended to saccade planning and working memory (here referred to as pupil local-luminance modulation). Although research in monkeys has further elucidated a central role of the frontal eye field (FEF) and superior colliculus in the pupil local-luminance modulation, their roles remain to be established in humans. Through applying continuous theta-burst transcranial magnetic stimulation over the right FEF (and vertex) to inhibit its activity, we investigated the role of the FEF in human pupil local-luminance responses. Pupil light reflex responses were transiently evoked by a bright patch stimulus presented during the delay period in the visual- and memory-delay tasks. In the visual-delay task, larger pupil constriction was observed when the patch location was spatially aligned with the target location in both stimulation conditions. More interestingly, after FEF stimulation, larger pupil constriction was obtained when the patch was presented in the contralateral, compared to the ipsilateral visual field of the stimulation. In contrast, FEF stimulation effects were absence in the memory-delay task. Linear mixed model results further found that stimulation condition, patch location consistency, and visual field significantly modulated observed pupil constriction responses. Together, our results constitute the first evidence of FEF modulation in human pupil local-luminance responses.

## Introduction

Pupil size changes constantly to regulate the amount of light projected onto the retina to optimize visual processing (Denton, 1956; Campbell and Gregory, 1960; Woodhouse and Campbell, 1975; Laughlin, 1992), as the pupil constricts after an increase in global luminance level (Loewenfeld, 1999; McDougal and Gamlin, 2015; May et al., 2019). Recently, there is a renewed interest in studying the pupil light reflex because a growing number of studies have demonstrated that this reflex response is modulated by high-level cognition (Steinhauer et al., 2000; Binda and Murray, 2015a; Mathôt and Van der Stigchel, 2015; Binda and Gamlin, 2017), providing an objective index for investigating various cognitive processes (e.g., Fabius et al., 2017; Hsu et al., 2020; Strauch et al., 2022).

Spatial attention, as one of the core cognitive functions, modulates the pupil light reflex response (Binda and Murray, 2015a; Mathôt and Van der Stigchel, 2015), for example, pupil light reflex responses evoked by a bright stimulus are greater when the location of the stimulus spatially overlaps with the locus of attention (referred to as the pupil local-luminance modulation). This attention-regulated modulation on pupil size is pronounced even when the global luminance is unchanged (Binda et al., 2013; Mathot et al., 2013; Naber et al., 2013; Mathôt et al., 2014; Binda and Murray, 2015b), and this effect has also been extended to saccade planning and working memory (e.g., Mathôt et al., 2015; Fabius et al., 2017; Unsworth and Robison, 2017). For example: pupil size is smaller during the planning of an eye movement to a stimulus in the bright background than in the dark background (Mathôt et al., 2015). Directly comparing the effects of saccade planning and working memory in the same study has further shown similar local-luminance modulations, that is, pupil size is smaller when the location of the bright patch, compared to the dark patch, is spatially aligned with the location prepared for an upcoming saccade or remembered in working memory (Wang et al., 2018).

The network of brain areas, including the frontal eye field (FEF), lateral intraparietal cortex (LIP), and superior colliculus (SC) have been causally implicated in the shifts of spatial attention and gaze (Wardak et al., 2004; Thompson and Bichot, 2005; Bisley and Goldberg, 2010; Krauzlis et al., 2013). The SC receives direct projections from the FEF and LIP (reviews: Wurtz et al., 2001; White and Munoz, 2011), and projects directly to the brainstem and the spinal cord to execute the orienting movement such as saccades (Scudder et al., 1996; Rodgers et al., 2006). Research in behaving monkeys has found larger pupil light reflex responses when a bright stimulus is presented at the location corresponding to FEF microstimulation (Ebitz and Moore, 2017). Through manipulating SC excitability via electrical microstimulation and lidocaine microinjection, research has further found that pupil size is altered according to local luminance level at the spatial location corresponding to the affected location in the SC map, implicating a causal role of the SC in the pupil local-luminance modulation (Wang and Munoz, 2018). Although these results in behaving monkeys suggest that the FEF and SC are causally involved in pupil local-luminance responses, the neural mechanisms of this modulation are yet to be examined in humans.

**FIGURE 1:**
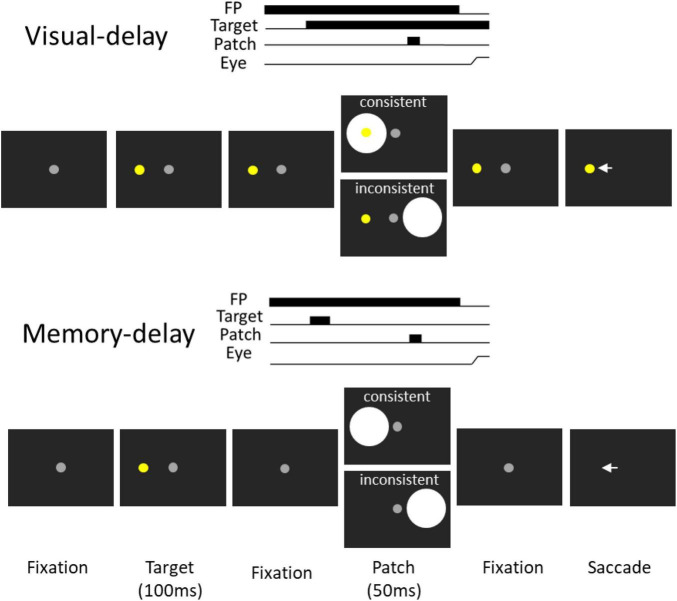
Experimental paradigm. Each trial started with a central fixation point on a black background. After a delay, there was a presentation of a target stimulus, and after a random delay the central fixation point disappeared and participants were required to move their eyes to the target. During the delay period, a bright circular patch stimulus was presented briefly (50 ms), with the patch being spatially aligned with the target location or the opposite location of the target in the consistent and inconsistent condition, respectively. Memory-delay task was similar to the visual-delay task except the target stimulus was only presented briefly (100 ms). Note that the figure only shows left-target conditions for illustration of the paradigm.

To investigate the functional role of the FEF in the pupil local-luminance modulation in humans, we applied continuous theta-burst transcranial magnetic stimulation (cTBS) over the right FEF and the vertex to disrupt the targeted regions using magnetic resonance imaging-guided transcranial magnetic stimulation (TMS), because the inhibitory effect associated with long-term depression lasting up to 1 hour has been observed after cTBS over the motor cortex (Huang et al., 2005), and we examined human pupil local-luminance responses in the visual- and memory-delay tasks (Figure 1). We hypothesized that the pupil local-luminance modulation should be observed in both visual-delay and memory-delay tasks, that is, larger pupil light reflex responses when the bright stimulus is presented at the location prepared for an upcoming saccade or remembered in working memory. More importantly, this pupil local-luminance modulation should be disrupted with FEF cTBS stimulation.

## Materials and methods

### Experimental setup

All experimental procedures were reviewed and approved by the Institutional Review Board of the Taipei Medical University, Taiwan, and were in accordance with the Declaration of Helsinki (World Medical Association, 2001). Twenty-eight healthy participants (8 males, mean age: 28.1, SD: 3.8 years) from Taipei Medical University were recruited, who were the same participants of another study (Hsu et al., 2021a). Sample sizes were chosen based on our previous studies with comparable pupillary and saccadic responses and trial numbers per participant (Wang et al., 2018; Hsu et al., 2020, 2021a; Cherng et al., 2021). Participants had normal or corrected-to-normal vision and were naïve regarding the purpose of the experiment. Participants provided informed consent and were compensated financially for their participation.

### Recording and apparatus

Participants were seated in a dark room. Eye position and pupil size were measured with a video-based eye tracker (Eyelink-1000 plus binocular-arm, SR Research, Osgoode, ON, Canada) at a rate of 500 Hz with binocular recording (left pupil was used), and stimulus presentation and data acquisition were controlled by Eyelink Experiment Builder. Stimuli were presented on an LCD monitor at a screen resolution of 1,920 × 1,080 pixels (60 Hz refresh rate), subtending a viewing angle of 58° × 32°, with the distance from the eyes to the monitor set at 60 cm.

### Theta-burst stimulation

To navigate the spatial location of targeted areas, T1-weighted images of MRI were acquired in each subject using 3T General Electric Discovery MR750 scanner with an 8-channel head coil. We carefully followed the well-established procedure for continuous theta-burst stimulation (Huang et al., 2005) that has been widely used for inhibitory effect (Huang et al., 2005; Gerits et al., 2011; Cameron et al., 2015; Cazzoli et al., 2015). Moreover, this protocol was also used to stimulate the right FEF in our previous research (Hsu et al., 2021a), and we expected to observe the inhibitory effects on the right FEF. Briefly, the cTBS pulses were administered with a Magpro X100 (MagVenture, Denmark) in a 70 mm figure-of-eight-shaped coil (MC-B70, MagVenture). Each cTBS session delivered a 40 s train of uninterrupted biphasic theta-bursts pulses. This consisted of 3 pulses, at 50 Hz, given in 200 ms intervals, comprising a total of 600 pulses for 40 s at 80% intensity stimulation for active motor threshold (AMT), which was applied over each brain region, as recommended by safety guidelines (Rossi et al., 2009). To determine 80% intensity stimulator output for the AMT, the motor evoked potential was elicited by placing the coil oriented 45° to the coronal plane and measured from the right first dorsal interosseous hand muscle using electromyography (MP160, BIOPAC). The AMT was defined as the lowest stimulator output in percentage that elicited 5 out of 10 twitches of more than 200 μV peak-to-peak amplitude in the contralateral hand, while the participant maintained 20% of a finger-thumb contraction (Huang et al., 2005). The mean AMT was 41.03% ± 6.15 (mean ± standard deviation) of maximum stimulator output.

The Talairach coordinates for the right FEF are [33 5.1 65] (Muggleton et al., 2003). Brainsight 2 (Rogue Research Inc., Canada) was used to navigate the spatial location of the FEF on participant’s head, and the vertex was manually measured and used as a control site. We stimulated the right FEF because we observed disrupted pupillary responses with right FEF cTBS (Hsu et al., 2021a). The participants came in twice (one week apart) for the same experiment with a different stimulation (the sequence of stimulation sites was counterbalanced across participants).

### Visual- and memory-delay saccade task

We used the visual-delay and memory-delay tasks because FEF delay activity is observed in these tasks in behaving monkeys (Sommer and Wurtz, 2000, 2001). The visual-delay and memory-delay patch paradigm (Wang et al., 2018) was modified to appropriately implement cTBS in the tasks. Participants were seated in a dark room and the experiment had 2 tasks (Figure 1 visual- and memory-delay) that were intermixed within a block of 335 trials lasting approximately 45 min. In the visual-delay task, each trial began with the appearance of a central fixation point (FP) (0.5° diameter; ∼10 cd/m^2^) on a black background (∼0.01 cd/m^2^). After a period (800–900 ms), a peripheral colored target (0.5° diameter; ∼45 cd/m^2^; referred to as the target stimulus) appeared to the right or left (radial angle: 0 or 180°) at an eccentricity of 7–9° visual angle from the central FP. After a variable delay (500–800 ms), a bright circular patch was displayed briefly for 50 ms (6° in diameter, ∼50 cd/m^2^, referred to as the patch stimulus). After another variable delay (1,200–1,350 ms), the FP was removed, and participants were required to generate a saccade toward the target. Two types of patch stimulus conditions were used (each condition had ∼20% of trials): in the consistent condition, the patch stimulus location was spatially aligned with the target location. In the inconsistent condition, the patch was presented in the mirror location of the target stimulus. In catch trials (∼10% of trials), no patch stimulus was presented, such that after a variable delay (500–800 ms) following the target onset, the FP was removed and participants were required to generate a saccade toward the target. In the memory-delay task, the configuration was identical to visual-delay configuration except that the target was only presented for 100 ms. Task condition (visual-delay or memory-delay), target location (left and right) and patch location (left and right) were randomly interleaved.

### Data analysis

Aspects of analyses related to time-on-task effects in the vertex stimulation condition have been published previously (Chen et al., 2022). Saccade reaction time (SRT) was defined as the time from fixation disappearance to the first saccade away from fixation (eye velocity exceeded 30°/s) with an amplitude greater than 3°. Trials were scored as correct if the first saccade after stimulus appearance was in the correct direction (toward the target). Failure to initiate a saccade within 1,200 ms after the disappearance of FP or with SRTs < 70 ms were considered as outliers and were excluded from analysis (<1% of trials). To maintain accurate measurement of pupil size around the patch presentation period, trials with an eye position deviation of more than 2° from the central FP or with detected saccades (>2° amplitude) during the period from 500 ms before to 1,200 ms after patch onset were excluded from analysis. When blinks were detected, following the literature, pre- and post-blink pupil values were used to perform a linear interpolation to replace pupil values during the blink period (Karatekin et al., 2010; Mathôt et al., 2018). Trials were discarded when two blinks occurred within a time interval of less than 500 ms.

Following the procedures of baseline-correction used previously (Bala and Takahashi, 2000; Moresi et al., 2008) for each trial, a baseline value was determined by averaging pupil size from 100 ms before to the appearance of the patch presentation. Pupil values were subtracted from this baseline value. To capture the peak pupil constriction response after the patch presentation, an epoch of 600–700 ms after the patch presentation was used (referred to as the peak epoch) because the time to peak constriction was ∼650 ms. Absolute pupil size (from 200 to 100 ms before patch onset) was also used to access tonic pupil size before the patch presentation.

A two way repeated-measure ANOVA was used to examine effects of patch-to-target consistency (consistent or inconsistent) and patch (or target) location (left or right) on the saccade or pupil response in FEF or Vertex stimulation. Bonferroni-corrected *t*-tests were used for the planned comparisons, except where indicated. A two-tailed student *t*-test was performed to compare the differences between the two conditions. Effect sizes (partial eta squared or Cohen’s d), where appropriate, are also reported. Statistical tests were performed using (JASP Team, 2019) and MATLAB (The MathWorks Inc., Natrick, MA, USA). Furthermore, following our previous pupil research (Cherng et al., 2021; Hsu et al., 2021a; Wang et al., 2021), we used a linear mixed model (LMM) to examine the impact of cTBS and other factors on the pupil constriction response that allowed us to include these variables as fixed effects while taking inter-participant variability into account (Pinheiro and Bates, 2000).

## Results

### Effects of frontal eye field-continuous theta-burst transcranial magnetic stimulation on saccadic reaction time

**FIGURE 2:**
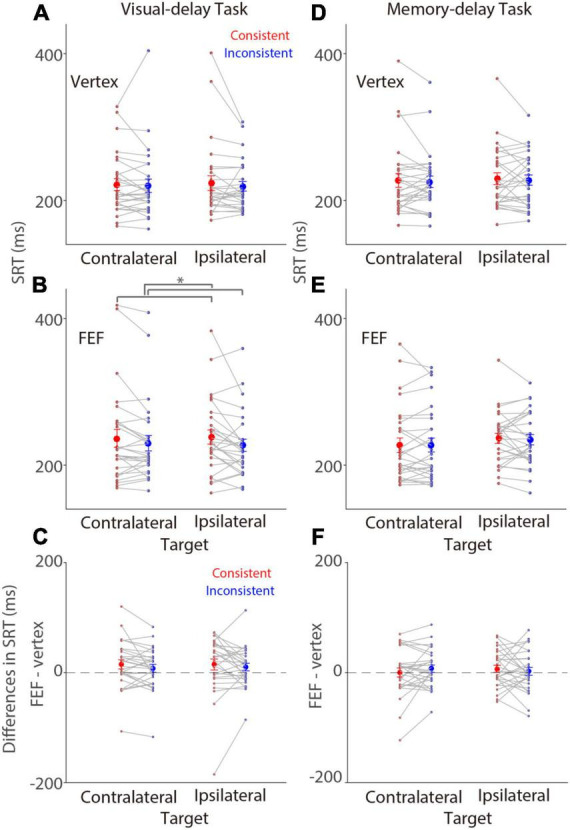
Modulation of FEF cTBS on saccade reaction time. Modulation of stimulation on SRTs (*N* = 28). **(A,B)** Mean values of SRTs with vertex **(A)** or FEF **(B)** stimulation in the visual-delay task. **(C)** Differences in SRTs between the contralateral and ipsilateral conditions (FEF minus vertex) in the visual-delay task. **(D,E)** Mean values of SRTs with vertex **(D)** or FEF **(E)** stimulation in the memory-delay task. **(F)** Differences in SRTs between the contralateral and ipsilateral conditions (FEF minus vertex) in the memory-delay task. The large circles and error-bars represent the mean values ± standard error across participants. The dots represent mean value for each participant. *Indicates differences are statistically significant (*p* < 0.05). Contralateral: contralateral stimulus condition (left). Ipsilateral: ipsilateral stimulus condition (right).

We first examined SRTs on trials with saccades to the left (referred to as contralateral) or the right (referred to as ipsilateral) target stimulus relative to stimulation (right FEF), that is, the patch and target location was spatially aligned or not aligned (referred to as consistency effects). In the visual-delay task, there was no consistency effect in SRTs with vertex stimulation [Figure 2A; *F*(1,27) = 0.826, *p* = 0.371, η_*p*_^2^ = 0.030]. The mean SRTs for contralateral-saccades (left-target) were 222 ± 8 (mean ± SEM) and 219 ± 6 ms in the consistent and inconsistent conditions, respectively, and 224 ± 10 and 220 ± 9 ms for ipsilateral-saccades (right-target). Effects of target location and interaction were also negligible (*p* > 0.6). In contrast, there was a significant consistency effect in SRTs with FEF stimulation, showing longer SRTs in the consistent condition [Figure 2B; *F*(1,27) = 6.715, *p* = 0.015, η_*p*_^2^ = 0.199]. The mean SRTs for contralateral-saccades were 236 ± 12 and 227 ± 8 ms in the consistent and inconsistent conditions, respectively, and 238 ± 9 and 230 ± 10 ms for ipsilateral-saccades. Other effects were negligible (*p* > 0.3). To directly examine the effects of FEF-cTBS, we contrasted SRTs between FEF and vertex stimulation. Figure 2C illustrates differences between FEF and vertex SRTs (FEF minus vertex), showing that SRTs were longer with FEF stimulation, particularly in the consistent condition. These results suggested that FEF cTBS seemed to increase SRTs, however, these effects were not significant (one sample *t* tests, all *p* > 0.079). Moreover, two way repeated-measure ANOVA (consistency & target visual field) showed no significant effects [visual field: *F*(1,27) = 0.063, *p* = 0.804, η_*p*_^2^ = 0. 002; consistency: *F*(1,27) = 1.873, *p* = 0.182, η_*p*_^2^ = 0.065; interaction: *F*(1,27) = 0.082, *p* = 0.777, η_*p*_^2^ = 0.003].

In the memory-delay task, there was no consistency effect in SRTs with vertex stimulation [Figure 2D; *F*(1,27) = 0.424, *p* = 0.521, η_*p*_^2^ = 0.015]. The mean SRTs for contralateral-saccades were 227 ± 9 and 228 ± 7 ms in the consistent and inconsistent conditions, respectively, and 230 ± 8 and 225 ± 8 ms for ipsilateral-saccades. Effects of target location and interaction were also negligible (*p* > 0.59). Similar results were observed with FEF stimulation (Figure 2E), the mean SRTs for contralateral-saccades were 227 ± 10 and 235 ± 7 ms in the consistent and inconsistent conditions, respectively, and 236 ± 7 and 227 ± 9 ms for ipsilateral-saccades. All effects were not significant (*p* > 0.13). Again, we contrasted SRTs between FEF and vertex stimulation to examine the effects of FEF-cTBS. Figure 2F illustrates differences between FEF and vertex SRTs (FEF minus vertex), showing similar SRTs between the two stimulation conditions (one sample *t* tests, all *p* > 0.28). Moreover, two way repeated-measure ANOVA (consistency & target visual field) showed no significant effects [visual field: *F*(1,27) = 0.048, *p* = 0.829, η_*p*_^2^ = 0. 002; consistency: *F*(1,27) = 0.116, *p* = 0.739, η_*p*_^2^ = 0.004; interaction: *F*(1,27) = 1.253, *p* = 0.273, η_*p*_^2^ = 0.044]. In summary, these results showed weak consistency effects on SRTs with FEF stimulation in the visual-delay task.

### Effects of continuous theta-burst transcranial magnetic stimulation and task on pre-stimulus tonic pupil size

**FIGURE 3:**
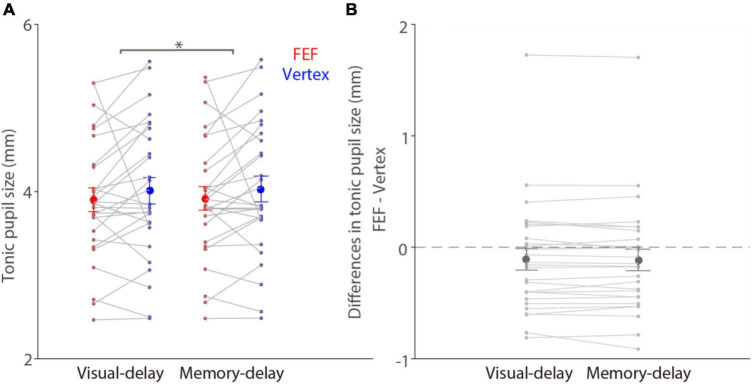
No modulation of FEF cTBS on tonic pupil size. **(A)** Modulation of stimulation on tonic absolute pupil size before the patch presentation (100–200 ms before patch onset) in the visual- and memory-delay tasks (*N* = 28). **(B)** Differences in tonic pupil size between the FEF and vertex stimulation conditions (FEF minus vertex). The large circles and error-bars represent the mean values ± standard error across participants. The dots represent mean value for each participant. *Indicates differences are statistically significant (*p* < 0.05).

Tonic (pre-patch absolute pupil size) and phasic (baseline-corrected) pupil responses are used to investigate different neural and cognitive processes (Aston-Jones and Cohen, 2005; Nassar et al., 2012; de Gee et al., 2014). To first examine whether FEF stimulation affected tonic pupil size, we analyzed absolute pupil size prior to the patch presentation between FEF and vertex stimulation (see section “Materials and methods”). Mean pupil sizes in the pre-patch epoch (100–200 ms before patch onset) for visual-delay trials were 3.9 ± 0.14 and 4.01 ± 0.16 mm in the FEF and vertex conditions, respectively, 3.92 ± 0.14 and 4.03 ± 0.15 mm for memory-delay trials (Figure 3A). Significantly larger pupil sizes were observed in the memory-delay task than in the visual-delay task [*F*(1,27) = 5.71, *p* = 0.024, η_*p*_^2^ = 0.175]. In contrast, FEF stimulation did not modulate tonic pupil size [*F*(1,27) = 1.37, *p* = 0.252, η_*p*_^2^ = 0.048], and the interaction was also not significant [*F*(1,27) = 0.61, *p* = 0.441, η_*p*_^2^ = 0.022]. Consistently, Figure 3B illustrates differences between FEF and vertex tonic pupil sizes (FEF minus vertex), showing statistically similar absolute pupil size between the two stimulation conditions (one sample *t* tests, all *p* > 0.24), though pupil size seemed to be numerically smaller with FEF stimulation. In summary, these results suggested that tonic pupil size was not reliably modulated by FEF stimulation.

### Effects of frontal eye field-continuous theta-burst transcranial magnetic stimulation on pupil light reflex responses evoked by patch stimuli

**FIGURE 4:**
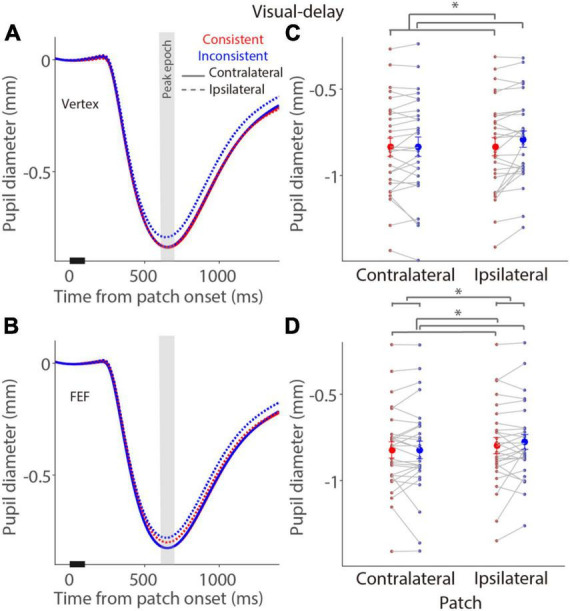
Modulation of FEF cTBS on pupil responses evoked by a patch stimulus in the visual-delay task. **(A,C)** Pupil dynamics in different conditions with vertex **(A)** or FEF **(C)** stimulation (*N* = 28). **(B,D)** Mean values of pupil responses (peak epoch: 600–700 ms after patch onset) with vertex **(B)** or FEF **(D)** stimulation. In panels **(A,C)**, the pupil response epoch is shaded in gray. The black bar on X-axis indicates the time line of patch presentation. In panels **(B,D)**, the large-circle and error-bars represent the mean values ± standard error across participants. The dots represent mean value for each participant. Contralateral: contralateral stimulus condition (left). Ipsilateral: ipsilateral stimulus condition (right).

To investigate whether pupil light reflex responses evoked by a bright patch stimulus during the delay period were modulated by the consistency between the patch and target locations in the visual- and memory-delay tasks (Figure 1), and whether these pupil responses were disrupted by FEF stimulation, baseline-corrected pupil size relative to patch onset was used (see section “Materials and methods”) to focus on the pupil light reflex responses (the peak pupil constriction response, see section “Materials and methods”) evoked by a patch stimulus during the delay period. As illustrated in Figure 4A, in the visual-delay task, pupil constriction was transiently evoked by patch stimuli in the vertex stimulation. Consistent with previous research (Wang et al., 2018), larger pupil constriction was observed when the patch location was spatially aligned with the saccadic target location [consistency main effect: *F*(1,27) = 8.877, *p* = 0.006, η_*p*_^2^ = 0.247], with mean pupil constriction sizes for the contralateral patch condition (left) in the peak epoch (600–700 ms after patch onset, Figure 4B) being –0.84 ± 0.052 and –0.83 ± 0.055 mm in the consistent and inconsistent conditions, respectively, –0.83 ± 0.052 and –0.79 ± 0.046 mm for the ipsilateral patch condition (right). Neither the patch visual field effect [*F*(1,27) = 3.347, *p* = 0.078, η_*p*_^2^ = 0.110] nor the interaction effect [*F*(1,27) = 2.927, *p* = 0.099, η_*p*_^2^ = 0.098] was significant. Similar pupil dynamics were observed with FEF stimulation (Figure 4C). Mean pupil constriction sizes for the contralateral patch condition in the peak epoch (Figure 4D) were –0.82 ± 0.046 and –0.82 ± 0.05 mm in the consistent and inconsistent conditions, respectively, –0.8 ± 0.046 and –0.78 ± 0.042 mm for the ipsilateral patch condition. Larger pupil constriction was obtained in the consistent than in the inconsistent condition [consistency main effect: *F*(1,27) = 4.704, *p* = 0.039, η_*p*_^2^ = 0.148], and larger pupil constriction was observed in the contralateral than in the ipsilateral patch condition [patch visual field main effect: *F*(1,27) = 18.433, *p* < 0.001, η_*p*_^2^ = 0.406]. No interaction was observed [*F*(1,27) = 0.498, *p* = 0.486, η_*p*_^2^ = 0.018]. These results suggested that pupil constriction was larger when the patch location was spatially aligned with the saccadic target location (consistency effects), and pupil constriction evoked by a patch stimulus was particularly disrupted with FEF stimulation (patch visual field main effects with FEF stimulation).

**FIGURE 5:**
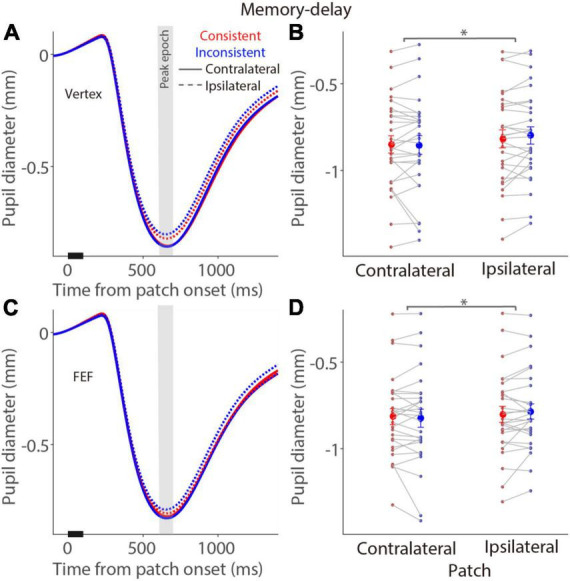
Modulation of FEF cTBS on pupil responses evoked by a patch stimulus in the memory-delay task. **(A,C)** Pupil dynamics in different conditions with vertex **(A)** or FEF **(C)** stimulation (*N* = 28). **(B,D)** Mean values of pupil responses (peak epoch: 600–700 ms after patch onset) with vertex **(B)** or FEF **(D)** stimulation. In panels **(A,C)**, the pupil response epoch is shaded in gray. The black bar on X-axis indicates the time line of patch presentation. In panels **(B,D)**, the large-circle and error-bars represent the mean values ± standard error across participants. The dots represent mean value for each participant. Contralateral: contralateral stimulus condition (left). Ipsilateral: ipsilateral stimulus condition (right).

In the memory-delay task, similar pupil constriction dynamics were observed after a patch stimulus (Figure 5). In the vertex stimulation condition (Figure 5A), mean pupil constriction sizes for the contralateral patch condition in the peak epoch (Figure 5B) were –0.85 ± 0.049 and –0.85 ± 0.053 mm in the consistent and inconsistent conditions, respectively, –0.82 ± 0.051 and –0.8 ± 0.049 mm for the ipsilateral patch condition. Larger pupil constriction was observed in the contralateral patch condition than in the ipsilateral patch condition [*F*(1,27) = 9.924, *p* = 0.004, η_*p*_^2^ = 0.269], however, no consistency and interaction effects were obtained [consistency: *F*(1,27) = 2.426, *p* = 0.131, η_*p*_^2^ = 0.082; interaction: *F*(1,27) = 0.829, *p* = 0.371, η_*p*_^2^ = 0.030]. Similarly, there was larger pupil constriction in the contralateral patch condition than in the ipsilateral patch condition with FEF stimulation [*F*(1,27) = 4.832, *p* = 0.037, η_*p*_^2^ = 0.152, Figure 5C], with mean pupil constriction sizes for the contralateral patch condition in the peak epoch (Figure 5D) being 0.82 ± 0.045 and –0.82 ± 0.051 mm in the consistent and inconsistent conditions, respectively, –0.8 ± 0.045 and –0.79 ± 0.043 mm for the ipsilateral patch condition. All other effects were negligible (*p* > 0.28). These results suggested no effects of FEF stimulation in the memory-delay task, and a general bias of larger evoked pupil responses induced by the patch presented at the left visual field, which is consistent with documented pseudoneglect phenomenon, an attention bias towards the left visual field (Jewell and McCourt, 2000; Strauch et al., 2022).

**FIGURE 6:**
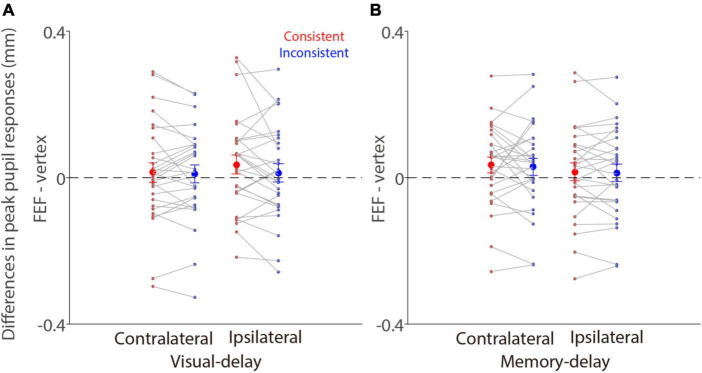
Effects of FEF cTBS between FEF and vertex stimulation conditions. **(A)** Differences in mean values of pupil peak responses (peak epoch: 600–700 ms after patch onset) between FEF and vertex stimulation (FEF minus vertex) in the visual-delay task (*N* = 28). **(B)** Differences in mean values of pupil peak responses (peak epoch: 600–700 ms after patch onset) between FEF and vertex stimulation (FEF minus vertex) in the memory-delay task (*N* = 28). The large-circle and error-bars represent the mean values ± standard error across participants. The dots represent mean value for each participant. Contralateral: contralateral stimulus condition (left). Ipsilateral: ipsilateral stimulus condition (right).

To directly examine the effects of FEF-cTBS, we contrasted pupil light reflex responses (peak epoch: 600–700 ms after patch onset) between FEF and vertex stimulation in the visual-delay and memory-delay tasks. Figure 6 illustrates differences between FEF and vertex pupil light reflex responses (FEF minus vertex), showing that pupil light reflex responses were reduced (less negative) with FEF stimulation, particularly in the consistent condition. These results suggested that, as predicted, FEF disruption reduced pupil light reflex responses evoked by patch stimuli, however, these effects were not significant (one sample *t* tests, all *p* > 0.11). Moreover, two way repeated-measure ANOVA (consistency & patch visual field) showed a marginally significant effect on the visual field in the visual-delay task [Figure 6A: visual field: *F*(1,27) = 3.645, *p* = 0.067, η_*p*_^2^ = 0. 119; consistency: *F*(1,27) = 1.784, *p* = 0.197, η_*p*_^2^ = 0.061; interaction: *F*(1,27) = 1.691, *p* = 0.204, η_*p*_^2^ = 0.059]. No significant differences were observed in the memory-delay task [Figure 6B: visual field: *F*(1,27) = 3.139, *p* = 0.088, η_*p*_^2^ = 0.104; consistency: *F*(1,27) = 0.249, *p* = 0.622, η_*p*_^2^ = 0.009; interaction: *F*(1,27) = 0.024, *p* = 0.877, η_*p*_^2^ = 0.001]. Together, we observed some disruptions in pupil light reflex responses induced by patch stimuli after FEF cTBS.

### Effects of stimulation, task type, consistency, and visual field on pupil light reflex using linear mixed model

To further examine the influence of these factors on pupil constriction responses, a linear mixed model was used that allowed us to consider data from all trials while taking inter-participant variability into account, because, as demonstrated, stimulation site, task type, patch-target location consistency, and patch visual field could all affect pupil light reflex responses induced by a bright patch stimulus. Our model included the dependent variable *y* (peak constriction size), stimulation condition, task type, patch-target location consistency, and patch visual field as fixed predictors. Following the standard approach, five models were used from the null model to the most saturated model based on our theoretical framework. Model comparison was performed based on AIC (Akaike information criterion) criterion. The linear mixed model was as follows:


(1) $$M\,o\,d\,e\,l\,1\;:\;y=\beta_{0}+\beta_{S}$$

(2) $$M\,o\,d\,e\,l\,2\;:\;y=\beta_{0}+\beta_{S}+\beta_{1}\,S$$

(3) $$M\,o\,d\,e\,l\,3\;:\;y=\beta_{0}+\beta_{S}+\beta_{1}\,S+\beta_{2}\,T$$

(4) $$M\,o\,d\,e\,l\,4\;:\;y=\beta_{0}+\beta_{S}+\beta_{1}\,S+\beta_{2}\,T+\beta_{3}\,C$$

(5) $$M\,o\,d\,e\,l\,5\;:\;y=\beta_{0}+\beta_{S}+\beta_{1}\,S+\beta_{2}\,T+\beta_{3}\,C+\beta_{4}\,V$$

where *S* is stimulation condition [FEF (1)/ vertex (2)], *T* is task type [visual- (1)/ memory-delay (2)], *C* is patch-target consistency condition [consistent (1)/ inconsistent (2)], *V* is patch visual field [left (1)/ right (2)], β_*S*_ is a Gaussian random variable fitted for each participant as an individual offset, and β_*i*_ are the standard coefficients of the statistical model (intercept and slopes). As shown in Table 1, Model 5 performed better than other models, and only the task type factor did not increase model performance. These results were reported in detail in Table 2, with the adjusted R-squared being 0.599. The regression coefficient for stimulation (β_1_) was –0.021 (β_1_ = –6.126, *p* = 9.27E-10), showing that smaller pupil constriction was observed with FEF stimulation, compared to, vertex stimulation. Moreover, patch consistency and visual field both significantly affected evoked pupil constriction responses (β_3_ = 9.478, *p* = 3.03E-21; β_4_ = 3.29, *p* = 1.00E-03), with larger pupil constriction correlating with the consistent and the contralateral (left) visual field patch condition. Task type effects were negligible (*p* > 0.15). Together, these results suggested that pupil constriction responses evoked by a patch stimulus were modulated by stimulation condition, consistency, and patch visual field.

**TABLE 1:** Akaike information criterion (AIC) for different models.

	AIC
Model 1	–5050
Model 2	–5086
Model 3	–5086
Model 4	–5094
Model 5	–5182

**TABLE 2:** Multilevel model for pupil constriction size.

	Regression coefficient	SE	*t*	*d.f.*	*P*
(Intercept)	–0.844	0.047	–18.007	13387	1.20E-71
Stimulation	–0.021	0.003	–6.126	13387	9.27E-10
Task	–0.005	0.003	–1.429	13387	1.53E-01
Consistency	0.032	0.003	9.478	13387	3.03E-21
Visual field	0.011	0.003	3.290	13387	1.00E-03

## Discussion

To understand the role of the FEF in the pupil local-luminance modulation in humans, we applied cTBS over the right FEF and the vertex, and examined pupil light reflex responses evoked by a patch stimulus presented at the spatial location prepared for saccades or remembered in working memory. In the visual-delay task, larger pupil constriction evoked by a patch stimulus was observed in the consistent than in the inconsistent condition with vertex stimulation. In the FEF stimulation condition, in addition to this consistency effect, larger pupil constriction was obtained in the contralateral (left) than in the ipsilateral (right) visual field condition. In contrast, in the memory-delay task, larger pupil constriction was observed in the left than in the right visual field condition irrespective of the stimulation condition. Importantly, LMM further showed that pupil constriction responses were modulated by stimulation condition, consistency, and patch visual field. Together, our results demonstrated FEF stimulation effects on pupil light reflex responses particularly in the visual-delay task, suggesting an involvement of the FEF in the control of human pupil local-luminance responses.

### Effects of frontal eye field continuous theta-burst transcranial magnetic stimulation on saccade reaction time in the visual- and memory-delay task

cTBS protocol has shown to effectively disrupt a targeted region lasting for up to 1 hour after stimulation (Huang et al., 2005). Therefore, we expected that SRTs, particularly in the contralateral field of stimulation, should be longer with FEF stimulation, compared to, vertex stimulation. In contrast, we only found statistically unreliable FEF stimulation effects particularly in the consistent condition in the visual-delay task, that is, longer SRTs were observed after FEF cTBS. But, it is important to note that effects of FEF stimulation on human SRTs are less reliable. Although some studies have found increased saccade latencies after right FEF stimulation (Nyffeler et al., 2006c,b,a), others have shown no effects on SRTs after FEF stimulation (Gurel et al., 2018; Hsu et al., 2021a). Future studies are certainly needed to examine the critical factors that determine FEF cTBS effects on SRTs.

### Pupil local-luminance effects between saccade planning and working memory

Pupil light reflex responses are modulated by spatial attention (Binda and Murray, 2015a; Mathôt and Van der Stigchel, 2015), with greater pupil constriction when the locus of attention spatially overlaps with a bright stimulus (or background), compared to a dark stimulus. Similar modulations are often obtained with saccade planning and working memory (Mathôt et al., 2015; Fabius et al., 2017; Unsworth and Robison, 2017, 2018; Wang et al., 2018). We thus expected that similar effects should be observed in the two tasks manipulating saccade planning and spatial working memory in the vertex stimulation condition. As predicted, with vertex stimulation, consistency effects were obtained in the visual-delay task, that is, larger evoked pupil constriction when the location of the bright patch was spatially aligned with the location of the target than when their locations were not aligned. However, these consistency effects were absent in the memory-delay task. Furthermore, larger pupil constriction was obtained in the left (contralateral) visual field condition in the memory-delay task. These results could be partly explained by a subtle leftward bias of spatial attention, a phenomenon often called pseudoneglect (Jewell and McCourt, 2000). More interestingly, a recent study has demonstrated that the pupil light reflex provides an objective measure of this bias (Strauch et al., 2022), showing larger pupil luminance effects when background luminance was changed on the left side, compared to the right side, of the central fixation point. Moreover, the differences in observed pupil responses between the two tasks could be explained by different attentional involvements. As demonstrated previously (Wang et al., 2018), although the pupil local-luminance modulation between the two tasks is similar, they are not identical. Specifically, pupil local-luminance modulations are diminished when there is no contingency implemented between the patch and target locations in the memory-delay task. Moreover, arousal effects between saccade planning and spatial working memory are also different. Overall, it seems that the local-luminance effects related to saccade planning are relatively more reliable. Moreover, to implement cTBS, the paradigm was modified to present the target only on the left or right of the FP (instead of 16 possible target locations with 8 different radial angles), and the catch trials (no patch) were also added. These changes could possibly reduce the consistency effects, particularly in the memory-delay task. Future research is certainly needed to address these questions.

### Neural mechanisms for pupil local-luminance effects between saccade planning and working memory

The FEF, LIP, and SC are causally involved in the control of gaze and attention shifts (Wardak et al., 2004; Thompson and Bichot, 2005; Bisley and Goldberg, 2010; Krauzlis et al., 2013). The SC receives critical control signals from the FEF and LIP (Wurtz et al., 2001; White and Munoz, 2011), and projects to the premotor brainstem circuit to initiate the orienting response including saccade and pupil responses (Wang et al., 2012; Corneil and Munoz, 2014; Wang and Munoz, 2015, 2021a; Lehmann and Corneil, 2016). This circuitry likely coordinates the pupil local-luminance modulation. Research in behaving monkeys has shown that pupil constriction is enhanced when a bright stimulus is spatially aligned with the corresponding location of FEF microstimulation (Ebitz and Moore, 2017). Furthermore, through changing SC excitability, research has revealed that the SC is causally involved in this pupil local-luminance modulation (Wang and Munoz, 2018). We thus expected that FEF cTBS should diminish pupil light reflex responses and disrupt consistency effects. Although pupil light reflex responses were reduced after FEF cTBS, compared to, vertex cTBS, these effects were not statistically significant. Moreover, dissimilar to our predictions, in the visual-delay task, there were still consistency effects with FEF stimulation (similar to the results observed with vertex stimulation). Interestingly, after FEF stimulation, pseudoneglect effects were observed with larger pupil constriction in the left (contralateral) than in the right (ipsilateral) visual field condition. Consistently, we found marginally significant visual field effects between FEF and vertex stimulation (*p* = 0.067), suggesting that FEF disruption affected attention mechanisms revealed by pseudoneglect effects in pupil light reflex responses. In contrast, in the memory-delay task, no effects were obtained except for the pseudoneglect effect of the patch visual field, showing that there was larger pupil constriction in the left (contralateral) than in the right (ipsilateral) visual field condition. Given that all these factors could affect pupil light reflex responses evoked by bright stimuli, LMM was used that included all influential factors while considering all trials and inter-participant variability. LMM results clearly showed that FEF cTBS, consistency, and visual field all significantly affected pupil constriction responses, particularly with reduced pupil light reflex responses after FEF cTBS.

In summary, our results suggested that FEF stimulation had some disruptive effects, though weak, on pupil light reflex responses, particularly in the visual-delay task, and these FEF effects were eliminated in the memory-delay task. FEF effects that only appeared in the visual-delay task are consistent with neuronal findings in comparing FEF activity between the visual- and memory-delay tasks in monkeys (Sommer and Wurtz, 2000, 2001; Wurtz et al., 2001), as FEF delay activity is more related to visual stimulation (visual-delay task). Therefore, it is possible that during the delay period, the FEF is particularly involved in the visual-delay task than in the memory-delay task, as a distributed network including frontal and parietal cortices is contributed to supporting working memory (MacKey and Curtis, 2017).

### Limitations and future directions

Pupil size is modulated by a great range of cognitive and affective processes (Loewenfeld, 1999; Eckstein et al., 2017; Einhäuser, 2017; van der Wel and van Steenbergen, 2018; Cherng et al., 2020; Eberhardt et al., 2021). As illustrated by LMM, here, we found that larger pupil constriction evoked by a patch stimulus correlated with FEF stimulation, patch inconsistency condition, and stimulation visual field condition. Although we did not observe a strong modulation of FEF stimulation on the pupil light reflex responses, as revealed by LMM results, evoked pupil constriction was indeed modulated by FEF stimulation. There are several reasons that could possibly explain these relatively small effects. First, we did not identify individual FEF location with functional MRI, as the gold standard. Instead, we used individual T1-image to navigate FEF location according to the standardized FEF coordinate. Thus, targeting FEF could be suboptimal in some participants due to individual differences in FEF location, resulting in weaker effects. Second, research has suggested that compensatory mechanisms (Sack et al., 2005) and changes in baseline activity (Goldsworthy et al., 2014) could be involved with the offline cTBS, together diminishing FEF stimulation effects. Future studies using the online TMS with the identification of FEF location following the gold standard are required to further address these possibilities. It is important to note that the FEF is also involved in the control of microsaccade generation (Peel et al., 2016; Hsu et al., 2021b), and microsaccade responses are modulated by various cognitive processes (Dalmaso et al., 2017, 2019) and correlate with pupillary responses (Wang et al., 2015; Dalmaso et al., 2020; Wang and Munoz, 2021b). Future studies that investigate microsaccade responses in the context of these modulations is needed to address these questions. A growing number of studies have used pupil size to understand human cognitive and affective processing, however, research that uses brain stimulation to understand the causal role of different brain areas in these pupil modulations is very limited (Hsu et al., 2021a). Further investigation using brain stimulation is thus critical to understand neural correlates of various pupil modulations.

## Data availability statement

The original contributions presented in the study are included in the article/supplementary material, further inquiries can be directed to the corresponding author.

## Ethics statement

The studies involving human participants were reviewed and approved by Institutional Review Board of the Taipei Medical University. The patients/participants provided their written informed consent to participate in this study.

## Author contributions

C-AW and T-YH designed the study. T-YH and H-YW performed the research. C-AW analyzed the data and drafted the manuscript. T-YH and J-TC provided the comments and edits on various drafts of the manuscript. All authors contributed to the study conception.
